# Pre-pandemic cognitive function and COVID-19 vaccine hesitancy: cohort study

**DOI:** 10.1016/j.bbi.2021.05.016

**Published:** 2021-08

**Authors:** G. David Batty, Ian J. Deary, Chloe Fawns-Ritchie, Catharine R. Gale, Drew Altschul

**Affiliations:** a Department of Epidemiology and Public Health, University College London, UK; b Lothian Birth Cohorts, Department of Psychology, University of Edinburgh, UK; c Department of Psychology, University of Edinburgh, UK; d Medical Research Council Lifecourse Epidemiology Unit, University of Southampton, UK

**Keywords:** Cognitive function, Cognitive ability, IQ, Mental ability, COVID-19, Vaccine hesitancy, Cohort

## Abstract

**Background:**

Whereas several predictors of COVID-19 vaccine hesitancy have been reported, the role of cognitive function is largely unknown. Accordingly, our objective was to evaluate the association between scores from an array of cognitive function tests and self-reported vaccine hesitancy after the announcement of the successful testing of the first COVID-19 vaccine (Oxford University/AstraZeneca).

**Methods:**

We used individual-level data from a pandemic-focused study ('COVID Survey'), a prospective cohort study nested within United Kingdom Understanding Society ('Main Survey'). In the week immediately following the announcement of successful testing of the first efficacious inoculation (November/December 2020), data on vaccine intentionality were collected in 11,740 individuals (6702 women) aged 16–95 years. Pre-pandemic scores on general cognitive function, ascertained from a battery of six tests, were captured in 2011/12 wave of the Main Survey. Study members self-reported their intention to take up a vaccination in the COVID-19 Survey.

**Results:**

Of the study sample, 17.2% (N = 1842) indicated they were hesitant about having the vaccine. After adjustment for age, sex, and ethnicity, study members with a lower baseline cognition score were markedly more likely to be vaccine hesitant (odds ratio per standard deviation lower score in cognition; 95% confidence interval: 1.76; 1.62, 1.90). Adjustment for mental and physical health plus household shielding status had no impact on these results, whereas controlling for educational attainment led to partial attenuation but the probability of hesitancy was still elevated (1.52; 1.37, 1.67). There was a linear association for vaccine hesitancy across the full range of cognition scores (p for trend: p < 0.0001).

**Conclusions:**

Erroneous social media reports might have complicated personal decision-making, leading to people with lower cognitive ability being vaccine-hesitant. With individuals with lower cognition also experiencing higher rates of COVID-19 in studies conducted prior to vaccine distribution, these new findings are suggestive of a potential additional disease burden.

## Highlights

• We used data from a large-scale, pandemic-focused, UK-wide cohort study.
• Lower cognition was robustly associated with COVID-19 vaccination hesitancy.
• Of concern, elsewhere, lower cognition is also related to higher COVID-19 rates.

## 1 Introduction

Cognitive function – also known as mental ability or intelligence – refers to psychological functions that involve the storage, selection, manipulation, and organisation of information, and the planning of actions (Deary, 2012, Deary and Batty, 2007). Assessed using standard tests, there is marked inter-person variation in how rapidly and precisely people carry out these mental tasks (Deary, 2012, Deary and Batty, 2007). Health protection and health care can also be regarded as a complex set of assignments that require assimilation of knowledge, decision-making, and planning. It has been posited that people with higher cognitive function manage preventative behaviours and treatment more effectively (Gottfredson, 2004), and there is growing evidence that this is the case.

In well-characterised cohort studies, relative to their lower-performing counterparts, people with higher ability are more likely to have a healthy diet (Batty et al., 2007b), choose dietary supplements (Whalley et al., 2003), and be physically active (Batty et al., 2007b). Those who score better on cognitive tests also have a lower probability of smoking cigarettes (Batty et al., 2007a, Batty et al., 2007c), drinking harmful levels of alcohol (Batty et al., 2006), and having associated problems (Batty et al., 2008). Cessation rates are also elevated in smokers with higher mental ability Taylor et al., 2005). Further, in individuals with a greater risk of a first cardiovascular disease event (Deary et al., 2009), in those with a higher probability of re-infarction,(Wallert et al., 2017) and in patients with respiratory disease (O'Conor et al., 2019), improved compliance with known efficacious drug therapies is apparent with higher ability scores. Similarly, in people with an elevated risk of colorectal cancer, rates of participation in a free screening programme were elevated in persons with better performance on tests of cognition (Gale et al., 2015).

These observations provide circumstantial evidence for a link between cognitive ability and another health-protecting behaviour, vaccine uptake. Vaccination is central to controlling the present pandemic, with success reliant on a sufficiently high uptake to achieve herd immunity (Omer et al., 2020). In the only empirical investigation of which we are aware, a very brief measure of analytical reasoning was administered to people in two small cross-sectional studies from the UK (N = 2025) and Ireland (N = 1041) (Murphy et al., 2021). Relative to the group who indicated they would be likely to accept a COVID-19 inoculation if one became available, somewhat lower cognition scores were apparent in study members indicating vaccine reticence (Murphy et al., 2021). These data were collected in March/April 2020 when no efficacious vaccine had been tested. Around 8 months later, following periodic statements to the media of its on-going development, successful testing of the Oxford University/AstraZeneca vaccine, the first known efficacious inoculation against COVID-19, was announced (Gallacher, 2020). Time-series analyses across multiple countries potentially implicates this declaration and those for other vaccines in having a positive impact upon intentionality (YouGov, 2021). Accordingly, for the first time to our knowledge, we investigated the link between cognitive function and COVID-19 vaccine hesitancy in a large UK general population-based sample in which data collection took place following the announcement of an efficacious vaccine.

## 2 Methods

**Fig. 1:**
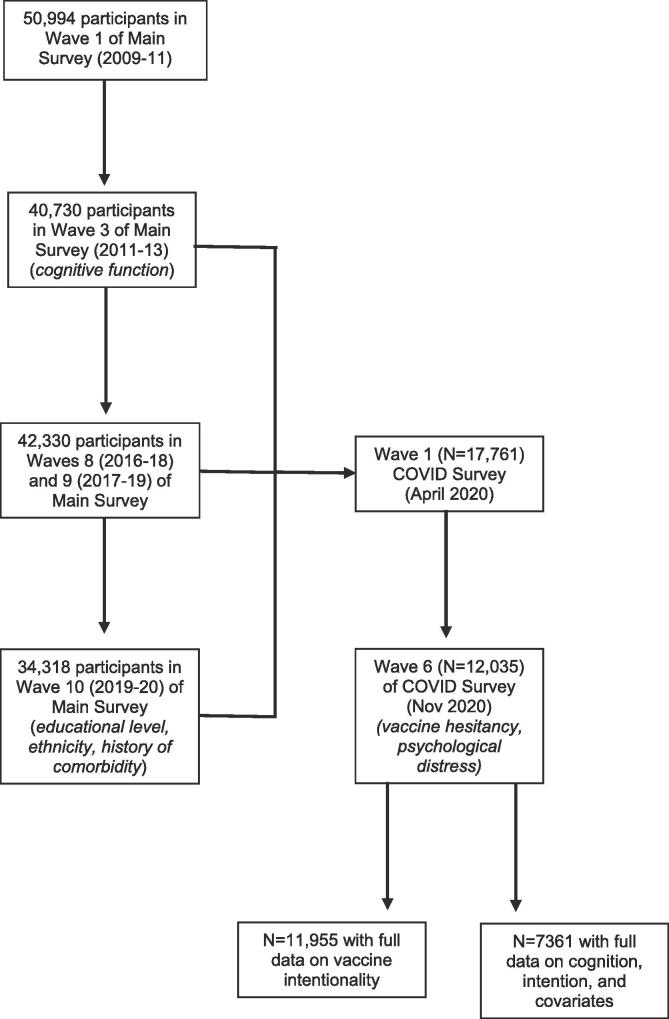
Flow of cohort members into the analytical sample: Main Survey and COVID Survey in Understanding Society

Understanding Society, also known as the United Kingdom Household Longitudinal Study, is a nationally-representative, on-going, open, cohort study (hereinafter, the ‘Main Survey’) (University of Essex, 2020). Based on a clustered-stratified probability sample of households, participants have been interviewed annually since 2009. Households who had participated in at least one of the two most recent waves of data collection (wave 8, 2016–18; wave 9, 2017–19) comprised the target sample for a pandemic-focused study initiated in April 2020 (hereinafter, the ‘COVID Survey’) (University of Essex, 2021). The derivation of the present analytical sample from the Main and COVID surveys, including the wave in which relevant data were collected, is depicted in Fig. 1. The University of Essex Ethics Committee gave approval for data collection in the COVID-orientated surveys (ETH1920-1271); no further ethical permissions were required for the present analyses of anonymised data.

The COVID Surveys took place monthly/bimonthly between April (wave 1) and, at the time of analyses, November 2020 (wave 6), with questions on vaccine intention first administered in the November wave when study members were aged 16–95 years (mean 53). Data collection in wave 6 (starting 24th November) commenced the day immediately following the announcement of the efficacy of the Oxford University/AstraZeneca vaccine (Gallacher, 2020) and continued for one week, comprising a total of 12,035 individuals of 19,294 invitations issued (response proportion, 62%).

### 2.1 Assessment of cognitive function

In the third wave of data collection in the Main Survey (2011–2013), six cognitive function tests were administered following piloting (Gray et al., 2011, McFall, 2013). Representing a range of cognitive skills, these tests have been repeatedly deployed in large-scale, population-based studies (Borsch-Supan et al., 2013, Lachman et al., 2010, Richards et al., 2004, Sonnega et al., 2014, Steptoe et al., 2013). Verbal declarative memory was assessed using both *immediate word recall* and *delayed word recall* tasks. Respondents listened to a list of ten words delivered by a computer; they were then asked to immediately recall the words and, again, at a later stage in the interview without having heard the words again. The number of correct responses was recorded on each occasion. For *semantic verbal fluency*, respondents named as many animals as they could in one minute; the final score was based on the number of unique correct responses. Using components of screening instruments for *cognitive impairment* including the Mini Mental State Examination (Crum et al., 1993) and the Cambridge Cognitive Examination (Huppert et al., 1995), respondents were asked to subtract 7 from 100 and then subtract 7 from their answer on four more occasions. The number of correct responses of a maximum of five was recorded. *Fluid reasoning* was assessed using a number sequence in which the respondent populated the gap(s) in a logical series. Respondents were initially presented with simple examples to test their understanding; those who seemed confused or unable to understand test requirements after the relaying of two examples were excused from the test. Remaining study members were administered two sets of three number sequences, with the difficulty of the second set determined by their performance on the first. A score was derived which accounts for the difficulty of the items. Lastly, for *numerical reasoning skills*, individuals were given three numerical problems to solve and, depending on their responses, were then administered a further one (simpler) or two (more difficult) problems. The total number of correct responses was recorded.

### 2.2 Assessment of covariates

Covariates were self-reported and included age; sex (both wave 10 [2019–20], Main Survey); ethnicity (wave 10, Main Survey; denoted as white or non-white); highest education level (wave 10, Main Survey; categorised as degree & other higher degree, A’ level or equivalent [Advanced Placement in the US], GCSE or equivalent [Grade 10 in the USA], other qualification, and none); and National Health Service-recommended shielding status for any household member (waves 1–5 [April-September 2020], COVID Surveys; denoted by yes/no). A history of physical morbidities was also captured (wave 10, Main Survey) and based on any mention of a cardiometabolic condition (congestive heart failure, coronary heart disease, angina, heart attack or infarction, stroke, diabetes, and/or hypertension); respiratory disease (respiratory disease comprised bronchitis, emphysema, chronic obstructive pulmonary disease, and/or asthma); or cancer of any type. Current psychological distress (wave 6 [November 2020], COVID Survey) was ascertained using the administration of the 12-item version of the General Health Questionnaire. Validated against standardised psychiatric interviews (Hankins, 2008, Holi et al., 2003), this is a widely used measure of distress (anxiety and depression) in population-based studies. Consistent with published analyses (Russ et al., 2011, Russ et al., 2015, Russ et al., 2012), we used a score of ≥ 3 to denote psychological distress.

### 2.3 Assessment of vaccine intentionality

At wave 6 in the COVID Survey, study members were asked “Imagine that a vaccine against COVID-19 was available for anyone who wanted it. How likely or unlikely would you be to take the vaccine?”. Possible responses were “Very likely”, “Likely”, “Unlikely” and “Very unlikely”. The latter two categories were combined to denote vaccine hesitancy.

## 3 Statistical analyses

It is well-replicated that performance on tests of cognitive abilities are positively inter-related, whereby people who score highly on one test of cognition tend to score well on another (Deary, 2012). This has led to the use of the term ‘general cognitive ability’, usually known as ‘g’. Accordingly, using scores from the six tests of cognitive function we generated a single general cognitive function variable. Computed using principal components analysis, the first unrotated component of the six cognitive tests was used as a single measure of cognitive function (variance explained: 42%; loadings: immediate recall 0.74, delayed recall 0.72, verbal fluency 0.59, serial sevens 0.49, number series 0.64, numerical problem solving 0.66). To summarise the relation between cognition and vaccine hesitancy, we used logistic regression to compute odds ratios with accompanying 95% confidence intervals. In these analyses we calculated effect estimates for tertiles of cognitive function scores (highest was the referent group) and those for a unit (standard deviation) disadvantage in score. The most basic analyses were adjusted for age, sex, and ethnicity. Retaining these covariates, we then explored the impact of separately controlling for existing somatic medical conditions, mental health, education, and shielding status.

## 4 Results

In a sample of 11,955 individuals who responded in full to the enquiry regarding COVID-19 vaccine intentionality, 17.2% (N = 1842) indicated that they were hesitant. In Table 1 we show unadjusted study member characteristics according to vaccine intention. Relative to the group who indicated a willingness to have the vaccine, those who were hesitant were more likely to be young, female, from an ethnic minority background, and be less well educated. The hesitant were also less likely to carry an array of existing somatic morbidities and be shielding or live with someone who was. The prevalence of psychological distress was somewhat higher in the vaccine hesitant.

**Table 1:** Study member characteristics according to COVID-19 vaccine hesitancy in Understanding Society

	Vaccine hesitant		P value for difference
	Yes	No	
Numbers of people (%)	1842 (17.2)	10,113 (82.8)	-

Demographic factors			
Age, yr, mean (SD)	45.0 (14.5)	54.6(15.6)	<0.0001
Female, N (%)	1162 (63)	5530 (55)	<0.0001
Non-white ethnicity, N (%)	406 (22.0)	698 (7.0)	<0.0001

Socioeconomic factors			
No university education, N (%)	939 (51.0)	4298 (42.5)	<0.0001

Comorbidities			
Cardiometabolic disease, N (%)	268 (15.0)	2513 (25.2)	<0.0001
Respiratory disease, N (%)	219 (12.3)	1372 (13.8)	0.144
Any cancer, N (%)	45 (2.5)	525 (5.3)	<0.0001
High psychological distress, N (%)	509 (27.6)	2399 (23.7)	<0.0001
Shielding in the household, N (%)	196 (10.6)	1187 (11.7)	<0.0001

**Cognitive function**			
*g* factor, mean (SD)	96.6 (15.7)	100.5 (14.8)	<0.0001

Numbers of study members corresponds to those with complete data

on vaccine intentionality

There were also differences in cognitive function between the vaccine groups, whereby the vaccine hesitant study members had lower general ability scores (difference in mean score 3.9; p-value for difference: <0.0001). We investigated these differentials in more detail in Table 2 where we present the results of regression analyses incorporating potential explanatory variables in an analytical sample of 7361 people with full data for all the variables depicted; that is, a non-missing dataset (Fig. 1). In age-, sex- and ethnicity-adjusted analyses, a one standard deviation lower score in general cognitive ability was associated with a 76% greater risk of being vaccine hesitant (odds ratio; 95% confidence interval: 1.76; 1.62, 1.90). While separate adjustments for somatic comorbidity, psychological distress, and shielding had no impact on this relationship, adjustment for education led to some attenuation (1.52; 1.37, 1.67) – the Kendall rank correlation between cognition and educational attainment was 0.27 (p < 0.0001). Simultaneous adjustment for all covariates had no greater attenuating effect than the adjustment for education alone.

**Table 2:** Odds ratios (95% confidence interval) for the relation of general cognitive function with COVID-19 vaccine hesitancy in Understanding Society

	Number hesitant/Total at risk	Age, sex, & ethnicity	Age, sex, ethnicity, & somatic comorbidity	Age, sex, ethnicity, & psychological distress	Age, sex, ethnicity, & shielding	Age, sex, ethnicity, & education	All covariates
Tertile 3 (high)	236/2284	1 (ref)	1 (ref)	1 (ref)	1 (ref)	1 (ref)	1 (ref)
Tertile 2	352/2918	1.28 (1.07, 1.54)	1.29 (1.08, 1.55)	1.28 (1.07, 1.54)	1.29 (1.08, 1.55)	1.17 (0.98, 1.41)	1.18 (0.99, 1.42)
Tertile 1	365/2159	1.99 (1.66, 2.40)	2.01 (1.67, 2.43)	1.99 (1.66, 2.40)	2.01 (1.67, 2.42)	1.64 (1.35, 1.99)	1.67 (1.37, 2.03)
P for trend		p < 0.0001	p < 0.0001	p < 0.0001	p < 0.0001	p < 0.0001	p < 0.0001
Per SD decrease	953/7361	1.76 (1.62, 1.90)	1.77 (1.63, 1.91)	1.76 (1.62, 1.90)	1.78 (1.64, 1.91)	1.52 (1.37, 1.67)	1.54 (1.40, 1.69)

Numbers of study members in this sample corresponds to those with complete data on all variables in the analyses. Thresholds for categories of cognitive function: tertile 1 (>=108.3); tertile 2 (108.2–93.3); and tertile 1 (>=93.2). A standard deviation (SD) in general cognitive function was 15 units.

To gain insights into whether the association with vaccine hesitancy was linear, the analyses were repeated according to tertiles of cognitive function. In age-, sex- and ethnicity- adjusted analyses, relative to people in the highest-scoring cognition tertile, those in the lowest were twice as likely to be vaccine hesitant (1.99; 1.66, 2.40). People in the intermediate ability tertile had intermediate risk of hesitancy (1.28; 1.07, 1.54) such that an incremental effect was apparent across the cognition categories (p-value for trend: <0.0001). To explore the impact on loss to follow-up, we derived inverse probability weights. Repeating our main analyses, our results were essentially unchanged (Table 1a, appendix).

**Fig. 2:**
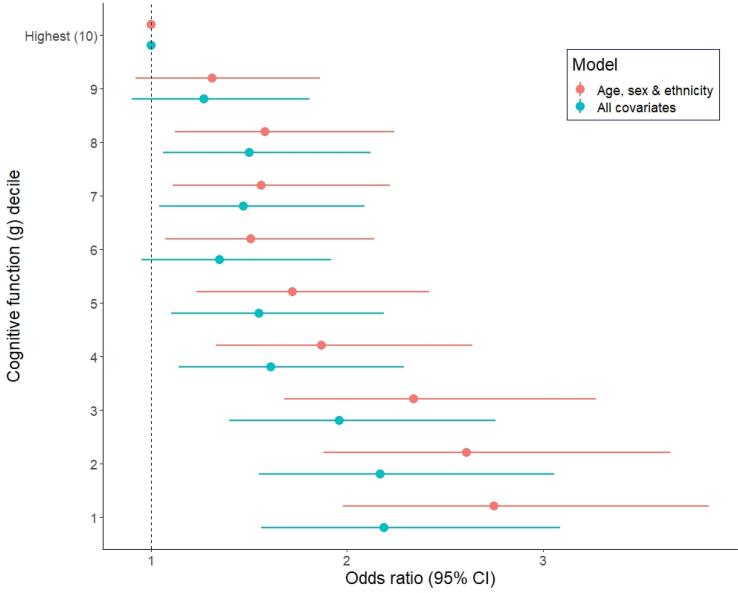
Odds ratios (95% CI) for the relation of general cognitive function with COVID-19 vaccine hesitancy in Understanding Society

Lastly, in order to explore inflections in the cognition–hesitancy association, we utilised deciles of cognition in analyses. Again, there was evidence of a clear trend, although this was not perfectly stepwise across all categories (Fig. 2).

## 5 Discussion

### 5.1 Principal findings

Our main finding was that, in data collected immediately following the announcement in the UK of an efficacious vaccine, and net of several covariates, people with lower scores on tests of cognitive function were less minded to take up an offer of vaccination for COVID-19 if it was made. These effects were apparent across the full range of cognition scores. That we were able to replicate known predictors of COVID-19 vaccine hesitancy – being female (Detoc et al., 2020, Freeman et al., 2020, Wang et al., 2020), younger (Detoc et al., 2020, Freeman et al., 2020), non-white ethnicity,(Freeman et al., 2020, Robertson et al., 2021, Williams et al., 2021), and having a *lower* prevalence of physical morbidity(Ruiz and Bell, 2021) – gives us some confidence in our novel results for cognitive function.

### 5.2 Comparison with existing studies

To the best of our knowledge, as described, there has been one prior examination of the relationship between cognition and vaccine hestinacy (Murphy et al., 2021). Comprising two small cross-sectional studies where data collection took place before the announcement of vaccine test results, people who performed better on a short test of cognitive function were more likely to be vaccine-accepting (Murphy et al., 2021). We found similar results in using more detailed measures of cognitive function in a large sample which allowed us to explore the shape of the relationship across the full range of abilities. A closely related literature is that for education with which cognition is positively correlated (Neisser et al., 1996). This supports the present results whereby people with higher educational achievement were less likely to be vaccine-hesitant (Kuter et al., 2021, Nguyen et al., 2021).

### 5.3 Potential public health implications

We have recently shown that, in data collected prior to vaccine distribution, of a range of baseline psychosocial factors which included socioeconomic status, education, personality type, and mental health, cognitive function was the most strongly and robustly associated with subsequent incidence of severe COVID-19, such that a doubling of the risk of hospitalisation was apparent in the lowest scoring group (Batty et al., 2020a). This supports other data that individuals with higher cognitive function experience a lower risk of death from other respiratory diseases, including influenza and pneumonia (Gale et al., 2019). The notion that people with lower cognitive ability appear to have greater rates of severe COVID-19 (Batty et al., 2021a, Batty et al., 2020a) and, based on the present results are also less likely to take up the offer of vaccination, may further increase the disease burden in this group, as may also be the case for people from ethnic minority groups (Lassale et al., 2020, Robertson et al., 2021) and the socioeconomically disadvantaged (Batty et al., 2020a, Murphy et al., 2021).

### 5.4 Plausible explanations

Various explanations may be germane to the cognition–vaccine intention link, including the observation that people with higher cognitive ability are better equipped to obtain, process, and respond to disease prevention advice (von Wagner et al., 2009). There has been a deluge of health advice in the current pandemic during an era when news outlets and social media platforms have never been more ubiquitous and influential. Preventative information has ranged from the simple and practical to the complex, contradictory, false, and fraudulent. In order to diminish their risk of the infection, people have to acquire, synthesise, weigh-up, and deploy this information but the ability to do so seems to vary by levels of health literacy (Wolf et al., 2020) just as it may for its close correlate, cognitive function.

### 5.5 Study strengths and weaknesses

While the present study has its strengths, including its size, national representativeness, and timing, there are also some weaknesses. First, we used vaccine intentionality as an indicator vaccine uptake but the correlation is imperfect. In a small scale longitudinal study conducted during the period of the 2009 H1N1 pandemic in Hong Kong, <10% of people who expressed a commitment to being inoculated reported that they had received a vaccination two months later (Liao et al., 2011). Elsewhere, in a US adult population at high risk of seasonal influenza, around half of those intending to be vaccinated had received the inoculation within the following 5 months (Harris et al., 2009). Second, there was inevitably some loss to follow-up (Fig. 1). While this attrition may have impacted upon the estimation of the prevalence vaccine hesitancy which is likely to be lower in our select sample relative to the general population (Fry et al., 2017), it is unlikely to have influenced our estimation of its relationship with cognitive function. Thus, in other contexts, we have shown that highly select cohorts reveal very similar risk factor–disease associations to those seen in studies with conventionally high response (Batty et al., 2020b). Also, as we have shown in sensitivity analyses, weighting had very little impact on the relation between cognitive function and vaccine hesitancy. Third, the 10 years period between cognitive testing and ascertainment of vaccine hesitancy raises concerns regarding the impact of changes in cognition function. While there was no retesting of cognition in the present study, findings from other studies suggest that cognitive function, even in older adults, is stable over a 10 year period, and, related, little mean decline will take place (Ritchie et al., 2019). Even less decline will have occurred in the present sample given that the age continuum covers all adulthood. There would also be modest inter-individual differences, such that people would largely retain their ranking from baseline. For context, all population characteristics are subject to some variation over time and cognition is no exception. As we have shown (Batty et al., 2021), in resurveys of samples of up to 31,000 individuals in UK Biobank, cognitive function scores (r = 0.63, p < 0.001, N = 9689) have comparable test–retest correlation coefficients compared with cigarette smoking (0.60, p < 0.001, N = 31,037), blood pressure (0.65, p < 0.001, N = 19,772), and diabetes (r = 0.63, P < 0.001, N = 31,037). As has been demonstrated (Clarke et al., 1999), this order of correlation may result in regression dilution that is likely to lead to underestimation of risk factor associations.

In conclusion, people with lower scores on standard tests of cognitive function reported being less willing to take up the future offer of vaccination for COVID-19. It is possible that erroneous social media news reports have complicated decision-making. Special efforts should be made to communicate clear information about vaccine efficacy and safety so that everyone—including those who report being less likely to choose vaccination—can make well-informed choices.

## Declaration of Competing Interest

The authors declare that they have no known competing financial interests or personal relationships that could have appeared to influence the work reported in this paper.
